# Internal Crack Initiation and Growth Starting from Artificially Generated Defects in Additively Manufactured Ti6Al4V Specimen in the VHCF Regime

**DOI:** 10.3390/ma14185315

**Published:** 2021-09-15

**Authors:** Carsten Wickmann, Christopher Benz, Horst Heyer, Kerstin Witte-Bodnar, Jan Schäfer, Manuela Sander

**Affiliations:** 1 Institute of Structural Mechanics, University of Rostock, Albert-Einstein-Str. 2, 18059 Rostock, Germany; christopher.benz@uni-rostock.de (C.B.); horst.heyer@uni-rostock.de (H.H.); manuela.sander@uni-rostock.de (M.S.); 2 Institute of Physics, University of Rostock, Albert Einstein-Str. 23-24, 18059 Rostock, Germany; kerstin.w.bodnar@gmail.com; 3 Leibniz Institute for Plasma Science and Technology, Felix-Hausdorff-Str. 2, 17489 Greifswald, Germany; jschaefer@inp-greifswald.de

**Keywords:** VHCF, FGA, ultrasonic fatigue, tension-compression, Ti6Al4V, EBM, artificially generated defects, additive manufacturing, FIB, cross section polishing

## Abstract

The aim of the present work was to investigate the ‘fine granular area’ (FGA) formation based on artificially generated internal defects in additively manufactured Ti6Al4V specimens in the early stage of fatigue crack growth in the ‘very high cycle fatigue’ (VHCF) regime. Fatigue tests were performed with constant amplitude at pure tension-compression loading (*R* = −1) using an ultrasonic fatigue testing setup. Failed specimens were investigated using optical microscopy, high-resolution ‘scanning electron microscopy’ (SEM), and ‘focused ion beam’ (FIB) techniques. Further, the paper introduces alternative proposals to identify the FGA layer beneath the fracture surfaces in terms of the ‘cross section polishing’ (CSP) technique and metallic grindings with special attention paid to the crack origin, the surrounding microstructure, and the expansion of the nanograin layer beneath the fracture surface. Different existing fracture mechanical approaches were applied to evaluate if an FGA formation is possible. Moreover, the results were discussed in comparison to the experimental findings.

## 1. Introduction

Crack initiation in the very high cycle fatigue regime for type II materials is frequently observed at non-metallic inclusions of different shapes and chemical compositions with a typical ‘fish-eye’ (FiE) formation. However, crack initiation also occurs at grain- or phase boundaries of the microstructure and processing defects such as porosities in the interior of components [1,2]. Under certain conditions, a characteristic area is formed around the crack initiating defect or structure during the fatigue loading. This area refers to ‘optical dark area’ (ODA) [3,4], ‘granular bright facet’ (GBF) [5], ‘rough surface area’ (RSA) or ‘fine granular area’ (FGA) [6] based on the identified characteristics or the microscopy method used. For Ti6Al4V the abbreviation ‘rough area’ (RA) is also frequently used [7,8]. However, the areas introduced by the different terms do not necessarily correlate with each other [9]. Accordingly, the size of an ODA or GBF, detected in optical microscopy and ‘scanning electron microscopy’ (SEM), respectively, does not necessarily correspond to the FGA, which refers to the existence of a nanograin layer below the fracture surface. According to the literature [10,11,12], at least a large part of the total lifetime (90% to 99%) during fatigue loading is assigned to the FGA formation, even though it is not clear whether the FGA is formed during crack growth [12,13,14,15] or crack initiation occurs at the size of the FGA [16,17,18], followed by crack growth in the fish-eye [10].

The FGA region shows some characteristic properties, for example, increased roughness on the fracture surface [19]. Further, the FGA size correlates with the applied load amplitude and with the fatigue life [12,19,20,21,22,23,24,25,26]. It is also assumed [5,6,27,28] that the cyclic stress intensity Δ*K*_FGA_, which is calculated at the border of the FGA, corresponds to the cyclic long crack growth threshold value Δ*K*_th,lc_. Thus, an FGA only appears, if the stress intensity factor of the defect is less than the threshold value [29,30]. Furthermore, it could be observed by means of FIB and ‘transmission electron microscope’ (TEM) investigations [6,26,31] that the microstructure directly beneath the fracture surface is affected and fine nanograins are generated in a thin layer for constant amplitude loadings, which differ from the basic microstructure. In the case of variable amplitude loadings, partial areas of fine nanograins were observed [26]. However, investigations for high-strength steel [31] and on Ti6Al4V [7] showed only FGA formations with negative *R*-ratios. Ritz et al. [26] illustrate that the FGA thickness beneath the fracture surface increases with decreasing stress ratio but above *R* = 0.1, no FGA formation was observed in [26], whereby Deng et al. [32] found fine-grained areas up to *R* = 0.3 [9].

The formation mechanism of the FGA in the vicinity of interior defects of type II materials is one of the most discussed unknowns in the ‘very high cycle fatigue’ (VHCF) regime. Furthermore, the main part of the fatigue life is attributed to the formation of this zone, which indicates the relevance for scientific research in this area. However, due to the fact that it is unknown at which inclusion or defect a crack will initiate, investigations of the FGA formation in conventionally manufactured specimens are hardly possible. ‘Additively manufacturing’ (AM) technologies such as the ‘electron beam melting’ (EBM) process offer the possibility to place a defect in the form of a sphere at a defined position inside a VHCF sample. Therefore, during the build process, internal defects were provided in the center of the measuring range. If the size and position of the artificial defect are well chosen, it is feasible to trigger crack initiation and propagation starting from the introduced defect in the interior of the specimen during fatigue loading. This could be observed in the investigated batch of Ti6Al4V VHCF samples. Moreover, the authors show, that an FGA can be generated in the interior of the EBM specimen in the vicinity of the artificially introduced defect. This was previously only shown for artificially induced surface defects in vacuum conditions by Spriestersbach et al. [33]. However, it is mentioned, that the paper neither focuses on the AM process properties nor on the built orientation and their influence on the fatigue properties of the investigated material.

The preparation of artificial defects in the interior of the specimen can provide a contribution to the understanding of the FGA formation in further studies. Moreover, to expose the nanograin layer or FGA beneath the fracture surface, the ‘cross section polishing’ (CSP) technique and the grindings of the cross sections are two promising approaches, because a much larger area can be examined in contrast to the common FIB investigations. This enables a much more effective investigation of the FGA formation.

## 2. Materials and Methods

### 2.1. Chemical and Mechanical Properties

**Figure 1:**
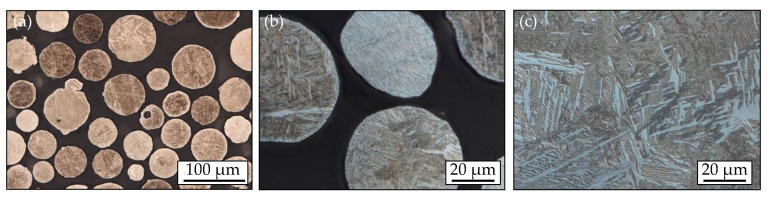
Grindings (**a**) of the Ti6Al4V granulate; (**b**) with zoom; (**c**) micrograph of the microstructure of an additive manufactured Ti6Al4V etched with Weck’s reagent.

The investigated material is the Ti6Al4V (3.7165) alloy with the mechanical properties listed in Table 1 and the chemical composition in Table 2. The EBM system ‘A1 by Arcam AB, Mölndal, Sweden’ was used for manufacturing the specimen by the ‘Chair of Microfluidics, University of Rostock’. The manufacturing process was carried out with the parameters according to [34] and the Ti6Al4V powder from Arcam AB with a mean particle size of 70 µm. Metallic grindings of the granulate used are shown in Figure 1a,b. Figure 1c, on the other hand, shows the microstructure of a sample after the manufacturing process.

### 2.2. Verification of Artificially Induced Defect Sizes and Position Using µCT Imaging

In order to verify the position and size of artificial defects in the measurement volume, ‘micro computed topography’ (μCT) investigations with ‘phoenix nanotom^®^ m-GE Inspection Technologies’ at the ‘Chair of New Materials, Institute of Physics, University of Rostock’ were carried out on 12 small cylindrical samples (Ø4 mm × 6 mm). Defect sizes of $\sqrt{A}$ = [100, 200, 300, 400] µm (sphere diameter *d* ≈ [113, 226, 339, 451] µm) were inserted into the cylinder samples during the design phase in the CAD model and exported as ‘standard tessellation language’ (STL) files for additively manufacturing with the EBM process. Therefore, the dimensions of standard defect types (pores and ‘lack of fusion’ defects) in additively manufactured materials from literature data [34,35] were selected as the basis for the defined defect sizes. The specimens were mounted on a rotary stage and scanned in their entirety, being rotated by 360° in 1400 equiangular steps. The detector size was 2284 pixels in x and y and 2304 pixels in the z direction. The voxel size of the images is 10.2 µm in all three axes.

**Figure 2:**
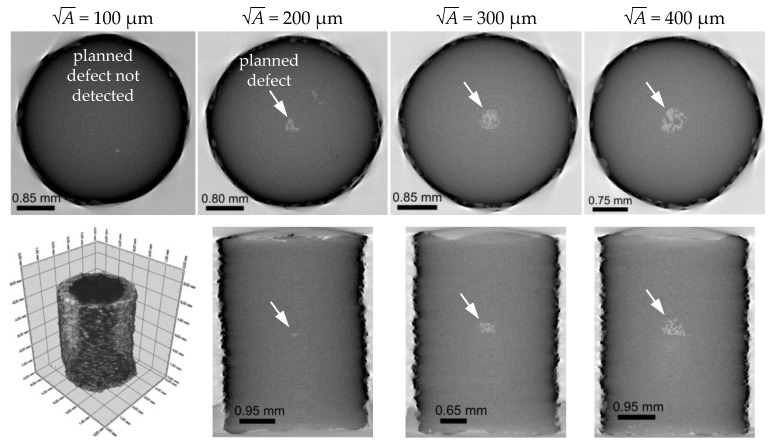
Verification of the size and the position of artificial defects in additively manufactured specimens of Ti6Al4V by means of µCT investigations in top and side view [36].

**Figure 3:**
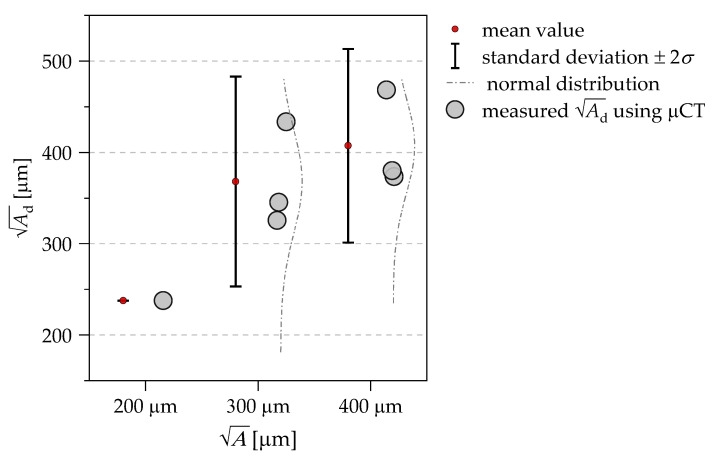
Evaluation of the target size by means of the $\sqrt{A}$ parameter using μCT imaging.

Figure 2 shows the position and the size of the detected defects listed for the respective $\sqrt{area}$ or $\sqrt{A}$ parameter. Furthermore, Figure 3 shows the results of the µCT investigations of the artificial defect sizes. For the smallest defect size of 100 µm, no clear defect could be detected at the planned position in all three examined cylinder samples. At 200 µm, only one sample showed a clear central defect with μCT imaging. For 300 µm and 400 µm, the planned defects were clearly identified. As the μCT investigations show, the cavities are filled with unmelted or partially pre-sintered powder.

### 2.3. Specimen Geometry and Test Procedure

**Figure 4:**
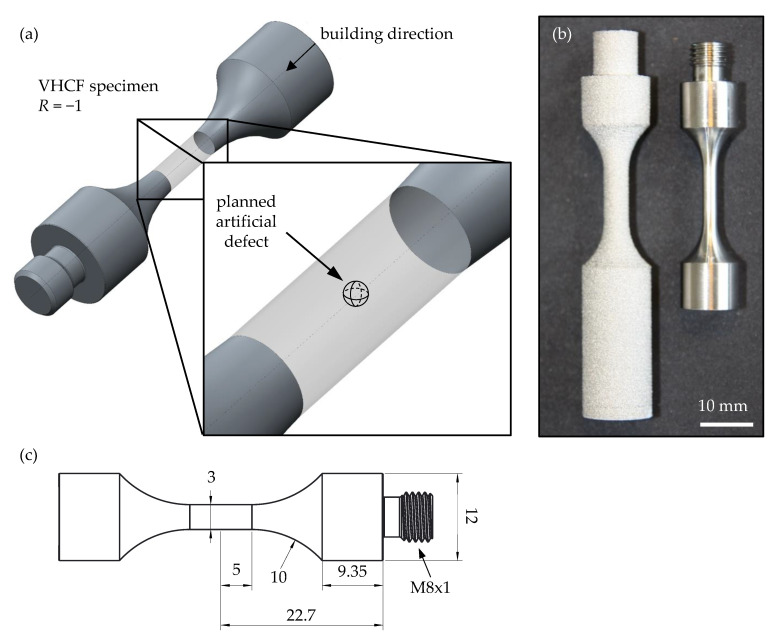
(**a**) Positioning of the artificial defect in the VHCF specimen; (**b**) view of EBM specimen ‘as built’ and after machining and polishing; (**c**) dimensions of the specimen after machining in mm. [36].

Based on the preliminary investigations, ultrasonic fatigue test samples were additively manufactured by the ‘Chair of Microfluidics, University of Rostock’ using the EBM system. Internal defects with $\sqrt{A}$ = [150, 200, 300] µm (sphere diameter of *d* ≈ [169, 226, 339] µm) were provided in the center of the measuring range of the ‘as built’ VHCF specimens during the manufacturing process as shown in Figure 4a. The specimens were machined after the building process to their final dimensions according to Figure 4b,c, and the cylindrical gauge length was mechanically polished with wet abrasive paper up to #4000 grit.

With these specimens, constant amplitude fatigue tests were performed with a stress ratio of *R* = −1 using the BOKU-Vienna fatigue testing equipment with the opportunity of a superimposed mean stress up to the limit number of cycles of *N*_f_ = 10^9^ cycles. To avoid self-heating of the specimens, all tests were carried out in pulse-pause mode with additional compressed air cooling. For detailed information concerning the fatigue testing system, refer to earlier publications [37,38,39]. If no fractures occurred after 10^9^ cycles, the specimens were repeatedly tested on higher load levels until fractures occurred.

## 3. Results

### 3.1. SN Data and Evaluation of Size and Positioning of the Artificial Defects

**Figure 5:**
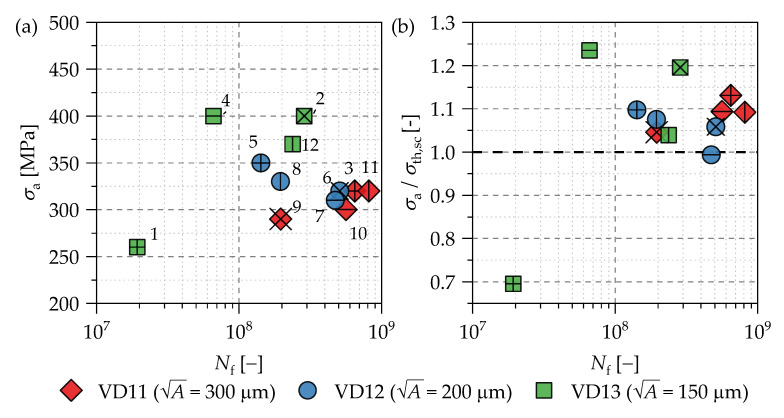
(**a**) SN data of EBM processed Ti6Al4V samples with artificially generated defects of different sizes and (**b**) normalized SN data with Murakami’s approach [4] according to Equation (1).

The results of the fatigue tests are shown in Figure 5a. Table 3 shows the test procedure of the marked specimen in Figure 5a, whereby the load steps and the number of repetitions due to reaching the limiting number of load cycles are given. For example, testing of specimen #2 started at a stress amplitude of 260 MPa and reached the limit of 10^9^ cycles on different load amplitude levels at 21 times, which sum up to 2.1 × 10^10^ cycles in total. Consequently, the stress amplitude, at which the sample failed was read as *σ*_a,f_ = 260 MPa + 12 × 5 MPa + 8 × 10 MPa.

All cracks initiate at the artificially generated internal defects inside the VHCF specimen, then grow firstly in the interior of the specimens until the surface is reached and then continue to spread semi-elliptically until a clear drop in the eigenfrequency of the specimen occurs (switch-off criterion of the ultrasonic testing machine). In Figure 5b, the stress amplitude is normalized by the threshold stress amplitude for short crack growth using Murakami’s approach [4] according to

(1) $$\sigma_{\mathrm{th},\mathrm{sc}}=\frac{1.56\cdot (HV+120)}{{\sqrt{A}}^{1/6}}$$

for internal defects. The $\sqrt{A}$ parameters were evaluated on the fracture surface based on the actual crack initiating defect sizes $\sqrt{A_{d}}$. With the exception of sample #1, the values of *σ*_a_/*σ*_th,sc_ for failed specimens are larger than ~1. Subsequently, the evaluation based on the $\sqrt{A}$ parameter model using Equation (1) predicts the fatigue limit for a fatigue life of 10^9^ cycles of the investigated dataset conservative.

**Figure 6:**
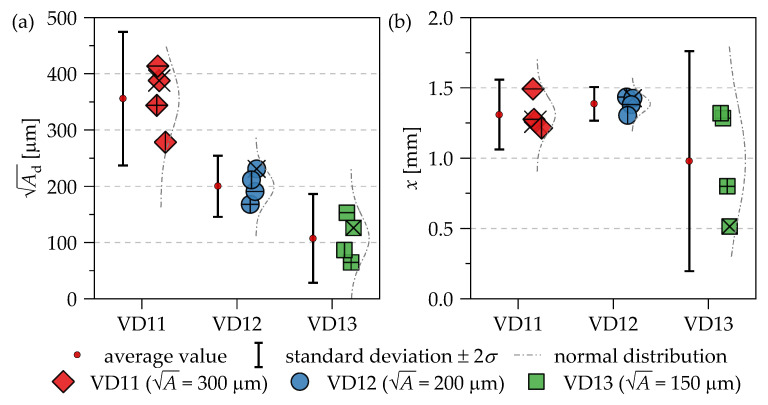
Evaluation (**a**) of the target size by means of the $\sqrt{A}$ parameter and (**b**) positioning of the artificial defects by measuring the distance from the specimen’s surface *x* on the fracture surfaces.

Figure 6a shows the evaluation of the measured defect sizes in terms of the $\sqrt{A_{d}}$ parameter. The average values, the distribution functions, as well as the standard deviations are plotted next to the data points. Compared to the nominal sizes, the mean values of the fracture-inducing defects tend to be larger for 300 µm and smaller for 150 µm. For 200 µm, a very good agreement between the mean values of the samples and the nominal value can be observed. Figure 6b shows the distance *x* from the surface to the defect for the three investigated defect sizes. With the exception of test series VD13 ($\sqrt{A}$ = 150 μm), the planned defect could be positioned very reliably in the center of the measurement volume (*x* = 1.5 mm). Moreover, for the samples with an artifact of $\sqrt{A}$ = 150 µm, it is partly not possible to identify a clear crack initiation location on the fracture surface, which is also reflected in the fatigue data of Figure 5a. In particular, one specimen of the VD13 series with an artificial defect of $\sqrt{A}$ = 150 µm fails at a very low-stress amplitude compared to all other samples of the test series. This leads to the assumption that the artificial defects with $\sqrt{A}$ = 150 μm are partially not clearly built up or melted in the EBM process. The evaluation of the artificial defects on the fracture surfaces in Figure 6 illustrates for the VD12 series with $\sqrt{A}$ = 200 µm a very accurate size in all samples and good positioning. Furthermore, the μCT investigations in Figure 3 show for $\sqrt{A}$ = 200 µm that the measured defect size is in the upper range of the standard deviation of Figure 6 for the VD12 series. For $\sqrt{A}$ = 300 µm, both the standard deviation and the mean value for μCT investigations and measured defect sizes on the fracture surfaces are in good agreement.

### 3.2. Fracture Surface Analyses

**Figure 7:**
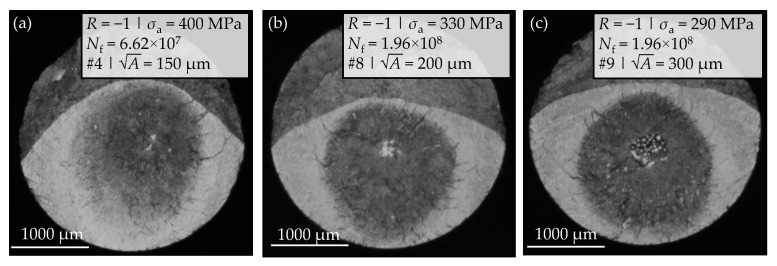
Comparison of fracture surfaces of VHCF specimen with artificially generated defects of (**a**) $\sqrt{A}$ = 150 μm; (**b**) $\sqrt{A}$ = 200 μm; (**c**) $\sqrt{A}$ = 300 μm [36].

The light microscopy images in Figure 7a–c show the fracture surfaces of selected samples with artificial defects. Characteristic dark areas are formed around the artificially introduced defects. As known from the literature, the RAs in titanium alloys [7,40] are larger compared to the GBFs or ODAs determined in steels [41]. Nevertheless, the overall dimensions of the investigated characteristic dark areas in the light microscopy images according to Figure 7 exceed the common RA sizes in conventional Ti6Al4 alloy [7,40].

**Figure 8:**
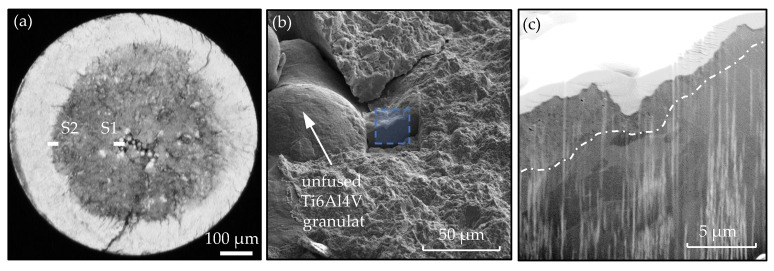
FIB investigations (performed at ‘Institute of Materials Science and Engineering, University of Kaiserslautern’) of the EBM processed VHCF specimen #3 with an additively manufactured defect of $\sqrt{A}$ = 300 μm at constant amplitude load and *R* = −1 (*σ*_a_ = 320 MPa, *N*_f_ = 6.51 × 10^8^). (**a**) optical micrograph of the positions of the FIB cuts; (**b**) zoom of FIB cut S1 at crack initiation location (SE, 10 kV); (**c**) view on the microstructure beneath the fracture surface with FGA formation (SE + In-Beam SE, 30 kV).

Moreover, FIB investigations were carried out in the vicinity of the artificially induced defect in order to investigate an FGA formation. Through the FIB section S1 in Figure 8 of a sample with an artificial defect of $\sqrt{A}$ = 300 μm, an FGA layer of 1 to 4 μm thickness can be observed directly at the crack initiation site. However, on a second FIB section (S2) at the transition area from the dark to the light region, no microstructural changes below the fracture surface were observed. This raises the question of whether the FGA is a continuous layer and how far it expands in the vicinity of the crack initiating defect.

### 3.3. Further Investigations for FGA Formation

FIB examinations for detecting the area of fine grains below the fracture surface are very expensive and time-consuming. In addition, only a very small local area can be investigated per section. In order to expose a larger area under the fracture surface and to investigate the change of the microstructure with regard to grain refinement, two proposals are presented in the following.

#### 3.3.1. Metallic Grinding and Etching

**Figure 9:**
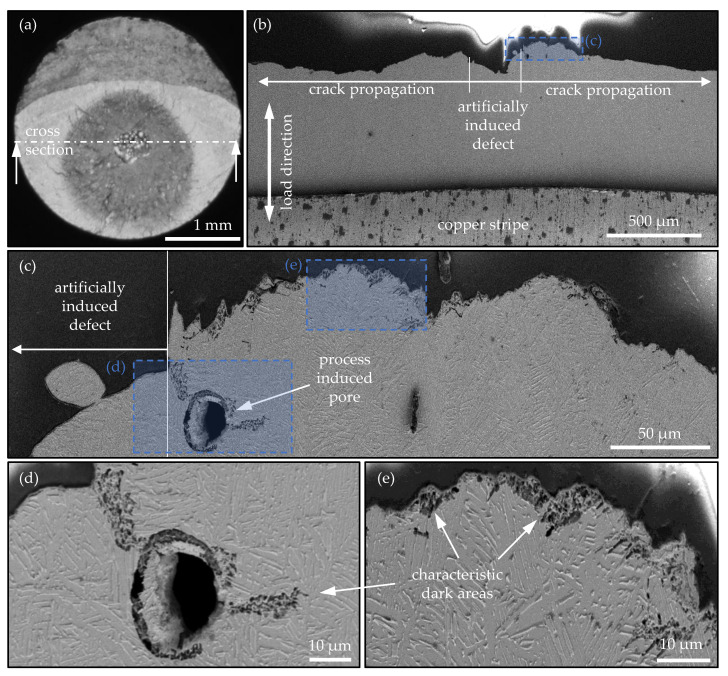
Investigation of a metallic grinding perpendicular to the fracture surface of the EBM processed VHCF specimen #9 with an additively manufactured defect of $\sqrt{A}$ = 300 μm. Etching of the metallic grinding with Kroll’s reagent (Metallic grinding and etching performed by the ‘Chair of Materials Science, University of Rostock’; SEM investigations performed at ‘Fraunhofer IGP Rostock’) (SE, 5 kV). (**a**) optical micrograph of the fracture surface with positioning of the cross section; (**b**) tilted SEM micrograph of the grinding with a view beneath the fracture surface; (**c**) magnification of the crack initiation location on the right-hand side of the artificial defect; (**d**) zoom in on a process-induced defect in the vicinity of the crack initiation location; (**e**) zoom beneath the fracture surface of the main crack.

The fracture surface in Figure 7c of specimen #9 was cut in the middle at the marked position and embedded as shown in Figure 9a. Subsequently, a metallic grinding was prepared and the sample was etched with Kroll’s reagent. Figure 9b shows an SEM image of the fracture surface profile of the sample of Figure 9a. The main crack propagates from the artificially introduced defect towards the outer surfaces of the specimen. The blue marked area can be seen enlarged in Figure 9c, where two more positions of the magnifications in Figure 9d,e are highlighted. Figure 9d shows a process-induced pore. Additionally, Figure 9e shows the microstructure below the fracture surface of the main crack. In both figures, characteristic dark areas can be observed partially under the fracture surface at the main crack. In the vicinity of the process-induced pore, the area can be determined continuously starting from the artificially introduced defect up to the pore. Subsequently, the dark structure appears around the pore and further propagates approximately 20 μm perpendicular to the load direction and then disappears. The thickness of the areas can be determined at approximately 5 μm, which correlates very well with the FIB investigations of Figure 7c in terms of FGA formation. If an FGA formation at the characteristic dark areas is assumed, changes in the microstructure of up to approximately 225 μm in distance to the end of the artificially induced defect can be observed according to Figure 9c.

**Figure 10:**
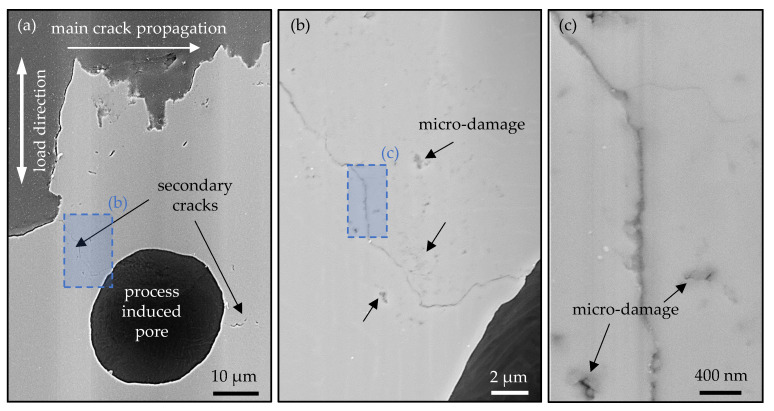
SEM investigation (performed at ‘Institute of Electronic Appliances and Circuits, University of Rostock’) of the polished metallic grinding perpendicular to the fracture surface of the EBM processed VHCF specimen #9 with an additively manufactured defect of $\sqrt{A}$ = 300 μm from Figure 9 (SE2, 10 kV). (**a**) process-induced pore in the vicinity of the crack initiation location; (**b**) zoom in on the marked position of (**a**) on a secondary crack; (**c**) zoom in the marked position of (**b**) with micro-damages in the vicinity of the secondary crack.

To verify whether the characteristic dark zones in the vicinity of the process-induced pore are accompanied by a crack, the mechanical section was subjected to further mechanical polishing to remove the traces of etching. Figure 10a shows an SEM image of the corresponding position. As the enlarged sections in Figure 10b,c show, the prominent zones in Figure 9d are followed by a secondary crack. In addition, micro-damages around the cracks with a size of about 0.2 μm could be observed (Figure 10c). These micro-damages also appear in the vicinity of the main crack below the fracture surface, as Figure 10a illustrates. Further, the micro-damages also were verified in the literature for example in the investigation of Sun et al. [42] in connection with FGA formation in martensitic stainless steel and of Su et al. [7] in connection with RA formation in Ti6Al4V alloy.

#### 3.3.2. Cross Section Polishing (CSP) Method

**Figure 11:**
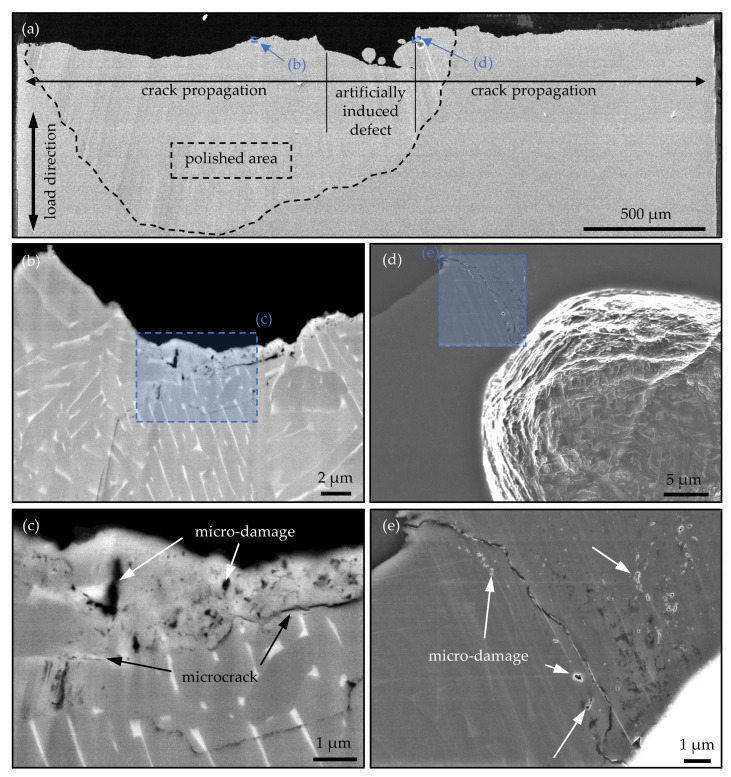
SEM investigation of the metallic grinding of Figure 10 of the EBM processed VHCF specimen #9 prepared by cross section polishing (CSP) process. (**a**) overview of the fracture surface (SEI, 15 kV); (**b**) zoom in the left marked area in (**a**) (LABE, 15 kV); (**c**) magnification of a position at the fracture surface with FGA formation (LABE, 15 kV); (**d**) zoom in the right marked area in (**a**) (SEI, 15 kV); (**e**) magnification of the secondary crack in the vicinity of the process-induced pore (SEI, 15 kV).

The mechanical section shown in Figure 10 was further prepared by cross section polishing at the INP in Greifswald and then investigated in the SEM. The sample was polished with a voltage of 6 kV for 8 h using an Ar+ ion beam of ‘Jeol IB-19530CP’. Using the CSP method, a much larger area (>500 μm [43]) below the fracture surface can be examined to investigate the microstructure, compared to FIB investigations. With the polished surface with little roughness, high-resolution examinations under the scanning electron microscope are then possible. Figure 11 shows the results of the CSP preparation. The polished area covers a distance of approximately 1.5 mm below the fracture surface and is further marked in Figure 11a. Figure 11b shows the enlargement of the marked position in Figure 11a with an LABE detector. As Figure 11b and the enlargement in Figure 11c illustrates, a partial change in the microstructure can be observed at the marked position, which is located roughly 360 μm in distance to the end of the artificially induced defect. Further, the figures show similar micro-damages and microcracks between the fine-grained structure and the coarse basic structure as compared to the studies of Sun et al. in [42].

Figure 11d,e shows the enlargement of the secondary crack in the vicinity of the process-induced pore with a standard SEI detector, which determines the microstructure to be not as pronounced in comparison to the LABE detector but shows more of the topographical events. Therefore, the LABE detector is preferred for grain refinement purposes. However, the micro-damages observed in Figure 10 could be shown clearly. If an FGA formation at the characteristic dark areas according to Figure 9 and at the microstructural changes in Figure 11 is assumed, the partial appearance can have the following reasons, among others. On the one hand, during specimen preparation and mechanical grinding, some of the zones were ablated, for example, due to poor adhesion of the embedding. Easy ablation compared to the basic structure is possible, because the formation of the FGA is often accompanied by micro-damages of the material and further microcracks between the fine-grained structure and the coarse basic structure [7,42]. On the other hand, following the FGA formation model by Sakai et al. [6], the crack forms exactly at the interface between the intact base material and the fine-grained zone.

### 3.4. Fracture Mechanical Approaches

**Figure 12:**
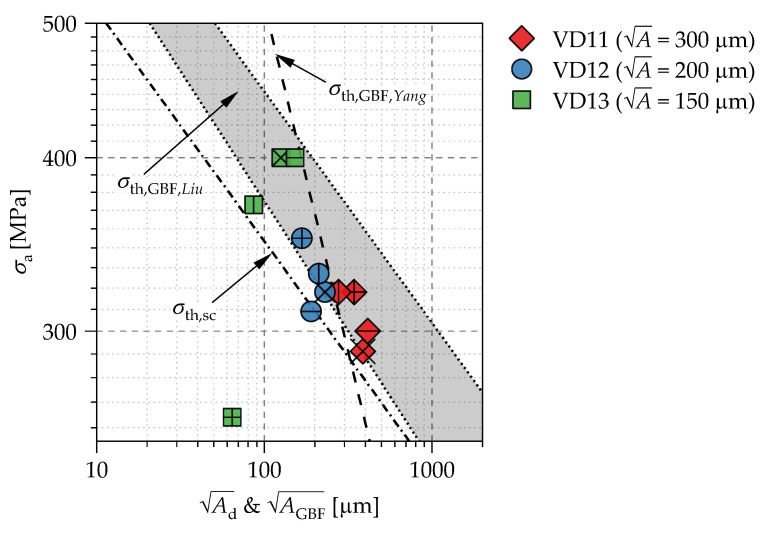
Modified Kitagawa–Takahashi diagram using the artificial defect sizes by means of the $\sqrt{A}$ parameter as crack length parameter.

Figure 12 illustrates a modified Kitagawa–Takahashi diagram using the $\sqrt{A}$ parameter as the crack length parameter. Therefore, the failure stress amplitudes are plotted versus the evaluated artificial defect sizes $\sqrt{A_{d}}$ of each sample.

For evaluating the crack initiation, the threshold stress value *σ*_th,sc_ by Murakami et al. [4] (Equation (1)) is plotted as a dashed-dot line in Figure 12. This curve shows that crack propagation is possibly starting from the artificial defects because the defect sizes result in stress amplitudes that are higher than the estimated threshold value according to the $\sqrt{A}$ concept.

Moreover, two concepts for FGA size estimation, which were designed for test data of different steels, according to the work of Yang et al. [25]:

(2) $$d_{\mathrm{GBF}}=\frac{2}{\sqrt{\pi }}\cdot \sqrt{A_{\mathrm{GBF}}}=1240\cdot \frac{1}{\sigma_{y}{}^{0.533}}\cdot \frac{1}{\sigma_{a}{}^{2}}\;\;\;\;\;\;\to \;\;\;\;\;\sigma_{\mathrm{th},\mathrm{GBF}}=\sqrt{\frac{620\cdot \sqrt{\pi }}{\sqrt{A_{\mathrm{GBF}}}\cdot \sigma_{y}{}^{0.533}\cdot 10^{-6}}}$$

with $\sqrt{A}$ in μm and *σ*_y_ im MPa as well as Liu et al. [41]:

(3) $$\sigma_{\mathrm{th},\mathrm{GBF}}^{H}=\frac{2\cdot \left(HV+120\right)}{{\sqrt{A_{\mathrm{GBF}}}}^{1/6}}$$

(4) $$\sigma_{\mathrm{th},\mathrm{GBF}}^{L}=\frac{2.7\cdot {\left(HV+120\right)}^{15/16}}{{\sqrt{A_{\mathrm{GBF}}}}^{3/16}}$$

with $\sqrt{A}$ in μm and *HV* in kgf/mm^2^ were applied for the titanium alloy and are shown in Figure 12. In both approaches, the GBF sizes instead of the granular layer beneath the fracture surface (FGA) in SEM micrographs were considered for the estimations.

It should be noted that the evaluated artificial defect sizes $\sqrt{A_{d}}$ and not the sizes of the characteristic zones (FGA, GBF, ODA, or observed characteristic dark areas of Figure 7 or Figure 9) were plotted in Figure 12. The experimental data appear close to the GBF size estimated by Equation (2) and the defect sizes of the VD11 series exceed the predicted values. Thus, an FGA formation especially for the VD11 series is unlikely, if the approach of Yang et al. [25] is applied, because of the large introduced defect sizes. However, a partial FGA was observed in specimen #9 of the VD11 series located roughly 360 μm in distance from the end of the artificially induced defect, which sums up to approximately $\sqrt{A_{FGA}}$ ≈ 1026 μm, if a full circle for FGA formation is assumed.

However, Liu et al. [41] propose a stress range delimited by two curves (Equations (3) and (4)) between which an FGA formation can be observed. If the applied stress amplitude is higher than the estimated threshold according to Equation (3), the FGA will not be formed. If the applied stress amplitude is lower than the estimated threshold according to Equation (4) the FGA is formed partially or not at all. About 75% of the tested specimen can be observed in the range estimated by the approach of Liu et al. [41], whereby most of the data points occur on the lower boundary. Thus, for the example of specimen #9, with the approach by Liu et al., a maximum GBF size of $\sqrt{A_{GBF}}$ ≈ 1409 μm according to Equation (3) is predicted, which is in better agreement with the measured value of $\sqrt{A_{FGA}}$ ≈ 1026 μm.

## 4. Conclusions

Artificially generated internal defects in different sizes were introduced in additively manufactured Ti6Al4V VHCF specimens. Using μCT imaging, the position and size of the internal defects were ensured. Constant amplitude fatigue tests were performed for *R* = −1 using the BOKU-Vienna fatigue testing equipment up to the limit number of *N*_f_ = 10^9^ cycles. All specimens were repeatedly loaded on higher load levels until fracture, with the aim to trigger fracture in the VHCF regime. The following conclusions can be drawn:

- μCT investigations were performed with cylindrical test specimens with introduced artificial defects of different sizes. Defect sizes with $\sqrt{A}$ ≥ 300 µm were observed in every case. For the samples with $\sqrt{A}$ ≤ 200 µm, this was only possible in one case.
- Crack initiation occurs at the artificial defects $\sqrt{A}$ ≥ 200 µm. Moreover, measurements of the defect sizes and their position on the fracture surfaces of the VHCF specimens illustrate a very reliable positioning in the center of the measurement volume.
- Investigations of the fracture surfaces with optical microscopy show rather large characteristic dark areas for the investigated specimens, which are formed around the artificially introduced defects.
- FIB preparations in the vicinity of the artificial defect show an FGA formation. A second FIB cut at the transition area from the dark to the light region shows no microstructural change below the fracture surface.
- In order to clarify the issue of whether the FGA is a continuous layer and how far it expands from the artificial defect, two methods were presented. Metallic grindings of the cross section area, as well as the cross section polishing method, provide two promising approaches to detect main crack topography and microstructural changes such as the FGA formation mechanism.
- In both preparation methods partial, microstructural changes were observed beneath the fracture surface in the vicinity of the artificially induced defect. However, also at a maximum distance of 360 μm from the end of the artificially induced defect, the characteristic dark area was observed by optical microscopy.
- A process-induced pore in the vicinity of the crack initiation location of the main crack was investigated. The results of the metallic grinding and the cross section polishing method investigated by SEM illustrate the same microstructural changes as those detected beneath the fracture surface of the main crack.
- Under the assumption that GBF and FGA correlate in size, two fracture mechanical approaches to estimate the GBF size were applied on the additively manufactured Ti6Al4V alloy. The artificial defect sizes on the fracture surfaces were evaluated by means of the $\sqrt{A}$ approach by Murakami [4]. The two criteria approach by Liu et al. [41] with an upper and a lower bound, where GBF formation is predicted, works well for the dataset.

## Figures and Tables

**Table 1:** Mechanical properties of the additive manufactured Ti6Al4V ^1^.

Material	*σ*_UTS_ [MPa]	*σ*_Y_ [MPa]	*E* [GPa]	*A*_5_ [%]	HV1 ^2^
3.7165	1120	1078	116	2.72	361

^1^ Tensile tests performed with VHCF specimen geometry. ^2^ Measurement of hardness at the ‘Chair of Materials Science, University of Rostock’. Mean value of six tests perpendicular and parallel to build direction.

**Table 2:** Chemical composition of the additive manufactured Ti6Al4V in weight-% ^3^.

Material	Ti	Al	V	Ni	Fe	Cu	Zr
3.7165	92.61	2.96	4.19	0.02	0.18	0.01	0.03

^3^ Chemical analysis via X-ray fluorescence spectroscopy at the ‘Leibnitz Institute for Catalysis’ in Rostock.

**Table 3:** Test procedure of the tested specimen.

Specimen	#1	#2	#3	#4	#5	#6	#7	#8	#9	#10	#11	#12
*σ*_a,1_ [MPa]	230	260										
∆*σ*_a_ [MPa]	10	5|10	10									
*i* [-]	3	12|8	6	14	9	6	5	7	3	4	6	11
$\sqrt{A}$ [μm]	150	300	150	200	300						150	
series	VD13	VD11	VD13	VD12	VD11						VD13	

## Data Availability

The data are not publicly available as further investigations are currently ongoing.
